# Hematologic and inflammatory parameters for determining severity of odontogenic infections at admission: a retrospective study

**DOI:** 10.1186/s12879-022-07934-x

**Published:** 2022-12-12

**Authors:** Junya Kusumoto, Eiji Iwata, Wensu Huang, Naoki Takata, Akira Tachibana, Masaya Akashi

**Affiliations:** 1 Department of Oral and Maxillofacial Surgery, Kakogawa Central City Hospital, Kakogawa, Japan; 2 Department of Oral and Maxillofacial Surgery, Kobe University Graduate School of Medicine, 7-5-2, Kusunoki-Cho Chuo-Ku, Kobe, 650-0017 Japan; 3 Department of Oral and Maxillofacial Surgery, Mitsubishi Kobe Hospital, Kobe, Japan; 4 Department of Oral and Maxillofacial Surgery, Hyogo Prefectural Awaji Medical Center, Awaji, Japan

**Keywords:** Cellulitis, Contrast-enhanced computed tomography, Deep neck abscess, Necrotizing soft tissue infection, Neutrophil-to-lymphocyte ratio, Systemic immune-inflammation index

## Abstract

**Background:**

Severe odontogenic infections in the head and neck region, especially necrotizing soft tissue infection (NSTI) and deep neck abscess, are potentially fatal due to their delayed diagnosis and treatment. Clinically, it is often difficult to distinguish NSTI and deep neck abscess in its early stage from cellulitis, and the decision to perform contrast-enhanced computed tomography imaging for detection is often a challenge. This retrospective case–control study aimed to examine the utility of routine blood tests as an adjunctive diagnostic tool for NSTI in the head and neck region and deep neck abscesses.

**Methods:**

Patients with severe odontogenic infections in the head and neck region that required hospitalization were classified into four groups. At admission, hematologic and inflammatory parameters were calculated according to the blood test results. In addition, a decision tree analysis was performed to detect NSTI and deep neck abscesses.

**Results:**

There were 271 patients, 45.4% in Group I (cellulitis), 22.5% in Group II (cellulitis with shallow abscess formation), 27.3% in Group III (deep neck abscess), and 4.8% in Group IV (NSTI). All hematologic and inflammatory parameters were higher in Groups III and IV. The Laboratory Risk Indicator for Necrotizing Fasciitis score, with a cut-off value of 6 and C-reactive protein (CRP) + the neutrophil-to-lymphocyte ratio (NLR), with a cut-off of 27, were remarkably useful for the exclusion diagnosis for Group IV. The decision tree analysis showed that the systemic immune-inflammation index (SII) of ≥ 282 or < 282 but with a CRP + NLR of ≥ 25 suggests Group III + IV and the classification accuracy was 89.3%.

**Conclusions:**

Hematologic and inflammatory parameters calculated using routine blood tests can be helpful as an adjunctive diagnostic tool in the early diagnosis of potentially fatal odontogenic infections. An SII of ≥ 282 or < 282 but with a CRP + NLR of ≥ 25 can be useful in the decision-making for performing contrast-enhanced computed tomography imaging.

**Supplementary Information:**

The online version contains supplementary material available at 10.1186/s12879-022-07934-x.

## Background

Cellulitis in the head and neck region is often encountered to varying degrees, and the disease has a good prognosis if treated appropriately [1]. Necrotizing soft tissue infection (NSTI) and deep neck abscess are rare and lethal. In the early stage, they are difficult to differentiate from cellulitis and thus, require special attention [2]. Early debridement and antimicrobial therapy are essential for NSTI. Vital signs and clinical findings such as the degree of swelling, respiratory distress, difficulty in opening the mouth, and painful swallowing are important for assessing the severity of the disease [3]. However, subjectivity cannot be avoided, thus reducing the accuracy of the assessment. The presenting symptoms of deep neck infections vary, with no definitive sign or symptom to distinguish the presence of drainable pus [4, 5].

Inflammatory markers identified by blood tests are commonly used as objective evaluation parameters, and C-reactive protein (CRP), white blood cell count (WBC), and its fractions (neutrophils, lymphocytes, monocytes) are often used as references. However, their values alone cannot determine disease severity. Computed tomography (CT) imaging, primarily contrast-enhanced CT (CECT), is considered essential for assessing patients with suspected gas production and abscess formation [6, 7]. However, it is difficult to apply to all cases of severe infections in the head and neck region.

The Laboratory Risk Indicator for Necrotizing Fasciitis (LRINEC) score has been proposed as an adjunctive diagnostic tool for NSTI [8], and we recently reported its usefulness [9]. Also, the neutrophil-to-lymphocyte ratio (NLR) and platelet-to-lymphocyte ratio (PLR) have been reported to be useful novel inflammatory biomarkers of infection [10, 11], including in head and neck infections [9, 12, 13]. Similarly, a new predictive marker, the systemic immune-inflammation index (SII), has recently been developed [14]; however, its application for infectious diseases has not been completely clarified.

This study aimed to investigate the utility of routine blood tests as an adjunctive diagnostic tool for the early detection of NSTI and deep neck abscesses in the head and neck region. We hypothesized that the combination of hematologic and inflammatory parameters calculated from the blood test data would make it possible to properly assess the severity of infection.

## Methods

### Study design and patients

This retrospective case–control study evaluated patients diagnosed with severe odontogenic infections of the head and neck region who required hospitalization at the Department of Oral Surgery, Kakogawa Central City Hospital (formerly Kakogawa East City Hospital) between January 2012 and March 2022.

The patients with severe odontogenic infection requiring hospitalization were included. The decision for hospitalization was based on the following criteria: abnormal vital signs and suspected sepsis; strong clinical findings of erythema, swelling, and heat sensation in the head and neck region; difficulty in eating or breathing; and the need for intensive intravenous antibiotic therapy. The exclusion criteria were as follows: age < 18 years, patients with tumors (solid and hematologic cancers), treatment with multiple intravenous antibiotics prior to the first visit, and missing laboratory data (WBC fractions, platelet, and the LRINEC score section described below).

Eligible cases were classified into four groups:

- Group I, cellulitis;
- Group II, cellulitis with superficial abscess formation (local onset, no spread into deep anatomical space);
- Group III, profound abscess formation (spread into deep anatomical spaces, deep neck abscess) [15];
- Group IV, NSTI.

Group II was defined as cases with clinical findings showing localized abscess formation and associated cellulitis. Group III was defined as cases with clinical findings and/or CECT showing abscess formation in deep anatomical spaces, along with final surgical confirmation of abscess formation. NSTI diagnosis was based on the diagnostic criteria of Fisher et al. [16] and Mathieu et al. [17] and confirmed by gas production findings on CT, intraoperative findings, and histopathology. CECT images were obtained when deep neck abscess or necrotizing fasciitis was suspected clinically. If the abscess had formed, incisional drainage was performed urgently and the drained pus was sent for bacterial culture. For NSTI, emergent debridement was performed and debrided necrotic tissue was sent for bacterial culture.

### Data collection

Data collection included age, sex, odontogenic causes, blood test data at admission, body temperature (˚C) at admission, body mass index, immunocompromised states (diabetes mellitus, using corticosteroids, and hemodialysis), the extent of opening mouth, admission to intensive care unit (ICU), duration of intravenous antibiotics administration, and duration of hospitalization (including treatment of the causative tooth). The following blood test data were also investigated on admission: CRP (mg/dL), WBC count (/µL) and its fractions (% neutrophils and lymphocytes), platelet (Plt) count (/µL), sodium (Na, mmol/L), creatinine (Cre, mg/dL), hemoglobin (Hb, g/dL), and blood glucose (Glu, mg/dL).

The LRINEC score, NLR, PLR, and SII were then calculated according to the data obtained from the blood tests. The LRINEC score was calculated as the sum of the scores for CRP, WBC, Hb, Na, Cre, and Glu [8]. SII was calculated as platelet count × NLR (× 10^4^) (a modified expression to emphasize NLR; original formula [platelet count × neutrophil count]/lymphocyte count). For SII, the order of magnitude was one order smaller than the original reference, which was adjusted to make the calculation more convenient [14]. Neutrophil left shift occurs early after infections [18], whereas there is a time lag in the production of CRP after initiation of infection [19]. This study calculated CRP + NLR (sum of CRP and NLR) as a new inflammatory marker to address the time lag in the production of CRP. The hematologic and inflammatory parameters, including CRP, WBC, NLR, PLR, SII, CRP + NLR, and the LRINEC score, were then compared among the four groups. Between Groups I + II and III + IV, the utility of the hematologic and inflammatory parameters was compared to identify the need for CECT imaging. Finally, a decision tree analysis was performed to find indicators to differentiate Group III + IV and to assist in decision-making for CECT imaging. Three researchers checked data individually (JK, EI, HW), and no discrepancies were found.

### Endpoints

The primary endpoint was evaluating the feasibility of using hematologic and inflammatory parameters as an adjunct to determine NSTI and deep neck abscesses in the head and neck region.

The secondary endpoint was identifying the characteristics of the severity of the different infections in the head and neck region.

### Statistical analysis

Representative values are presented as the median with range or the first and third quartiles. Fisher’s exact test was used for comparisons of nominal variables among the stages. The Brunner–Munzel test was used for two-group comparisons of continuous variables, and the Kruskal–Wallis test was used for multi-group comparisons. For testing trends among groups (Trend test), the Cochran–Armitage test was used for nominal variables, and the Jonckheere–Terpstra test was used for continuous variables. Cut-off values were determined from receiver operating characteristic (ROC) analysis using Youden’s index. To examine the usefulness of each parameter, the positive predictive value, negative predictive value, positive likelihood ratio, and negative likelihood ratio were also calculated. The decision tree analysis was performed by using the ‘rpart’ package. ‘rpart’ package is the machine learning library in R to build classification or regression models, and the resulting models can be represented as binary trees (https://cran.r-project.org/web/packages/rpart/index.html). All statistical analyses were performed using R software version 4.1.0 (R Development Core Team, 2021; R Foundation for Statistical Computing, Austria). The statistical significance was set at *P* < 0.05.

## Results

### Patient characteristics

Two hundred seventy-one patients with a median age of 61 years (range 18–103 years) were evaluated, with no difference in the proportions of males and females (50.9% versus 49.1%). The most common odontogenic cause was apical periodontitis (44.4%), followed by pericoronitis and osteomyelitis. Osteomyelitis was associated with bone destruction and included medication-related osteonecrosis of the jaw and osteoradionecrosis. It should be distinguished from apical periodontitis, periodontitis, and pericoronitis (the classification of the Japanese Society of Chemotherapy was used as reference material) [20]. There was an association between age and odontogenic causes. Pericoronitis was more common in younger patients (median 46 years, range 19–83), while periodontitis and osteomyelitis were more common in older patients (median 70 years, range 28–79; median 80 years, range 26–94) (Additional file 1: Fig. S1). The most common site was the mandibular molar region. Immunocompromised status was found in approximately a quarter of the patients, and all patients had used analgesics (non-steroidal anti-inflammatory drugs and acetaminophen) before visiting our department. The median duration of intravenous antimicrobial treatment was 7 days (range, 3–35 days) (Table 1).

**Table 1:** Characteristics of patients with severe odontogenic infections and comparison among the groups

	Patients (n = 271)	Group				*P* value	Trend test
		I (n = 123)	II (n = 61)	III (n = 74)	IV (n = 13)		*P* value
Age (years)	61 [41, 74.5]	56 [37, 71.5]	59 [40, 77]	65 [47.3, 73.8]	73 [41, 75]	0.087^a^	0.011^c^*
Sex						0.561^b^	0.971^d^
Male	138 (50.9%)	60 (48.8%)	35 (57.4%)	38 (51.4%)	5 (38.5%)		
Female	133 (49.1%)	63 (51.2%)	26 (42.6%)	36 (48.6%)	8 (61.5%)		
Body mass index	22.3 [20.1, 25.0]	22.3 [20.4, 25.1]	23.2 [21.2, 25.2]	21.3 [18.9, 24.8]	21.3 [18.7, 23.4]	0.036^a^*	0.064^c^
Cause						0.080^a^	0.274^d^
Apical periodontitis	120 (44.4%)	56 (45.9%)	36 (59.0%)	23 (31.1%)	5 (38.5%)		
Pericoronitis	36 (13.3%)	18 (14.8%)	5 (8.2%)	12 (16.2%)	1 (7.7%)		
Osteomyelitis	31 (11.5%)	12 (9.8%)	2 (3.3%)	14 (18.9%)	3 (23.1%)		
Post-extraction infection	23 (8.5%)	10 (8.2%)	2 (3.3%)	8 (10.8%)	3 (23.1%)		
Periodontitis	19 (7.0%)	5 (4.1%)	9 (14.8%)	4 (5.4%)	1 (7.7%)		
Others^†^	42 (15.5%)	22 (17.9%)	7 (11.5%)	13 (17.6%)	0		
Lesion						0.004^a^*	0.266^d^
Maxilla	54 (20.0%)	24 (19.7%)	23 (37.7%)	6 (8.1%)	1 (7.7%)		
Mandible	212 (78.2%)	96 (78.7%)	37 (60.7%)	67 (90.5%)	12 (92.3%)		
Others^‡^	5 (1.8%)	2 (1.6%)	1 (1.6%)	1 (1.4%)	0		
Location of odontogenic cause						0.005^a^*	0.403^d^
Anterior	33 (12.2%)	12 (9.7%)	14 (23.0%)	6 (8.1%)	1 (7.7%)		
Premolar	20 (7.4%)	8 (6.5%)	10 (16.4%)	2 (2.7%)	0		
Molar	192 (70.8%)	89 (72.4%)	33 (54.1%)	58 (78.4%)	12 (92.3%)		
Others^§^	26 (9.6%)	14 (11.4%)	4 (6.6%)	8 (10.8%)	0		
Compromised host^¶^	70 (25.8%)	22 (17.9%)	20 (32.8%)	21 (28.4%)	7 (53.8%)	0.011^b^*	0.006^d^*
Extent of opening mouth	30 [15, 40]	30 [15, 40]	40 [25, 40]	20 [15, 30]	25.0 [18, 29]	< 0.001^a^*	0.058^c^
Fever (°C)	37.2 [36.7, 37.7]	37.0 [36.6, 37.7]	37.2 [36.7, 37.6]	37.3 [36.6, 37.7]	37.4 [36.9, 37.8]	0.620^a^	0.401^c^
ICU	22 (8.1%)	1 (0.8%)	0	11 (14.9%)	10 (76.9%)	< 0.001^b^*	< 0.001^d^*
Duration of intravenous antibiotics (day)	3.5 [3.1, 4.0]	5 [4.3, 7]	6 [5, 8]	8 [7, 11.3]	12.5 [8.8, 15.3]	< 0.001^a^*	< 0.001^c^*
Duration of admission (day)	9.5 [5.9, 15.2]	7 [6, 9]	8 [7, 11]	14.0 [10, 18]	25.5 [16, 36]	< 0.001^a^*	< 0.001^c^*

Data are shown as the median [first quartile, third quartile] or n (%)

*ICU* intensive care unit

^*^Statistically significant (*P* < 0.05)

^a^Kruskal − Wallis test; ^b^Fisher’s exact test; ^c^Jonckheere − Terpstra test; ^d^Cochran − Armitage test

^†^Odontogenic maxillary sinusitis; sialadenitis; trauma; surgical site infection; odontogenic cyst; foreign body

^‡^Buccal; tongue^§^, maxillary sinus; salivary gland; lip^¶^, diabetes mellitus; corticosteroids usage; hemodialysis

### Characteristics, laboratory parameters, and pathogenic bacteria for each group

**Fig. 1:**
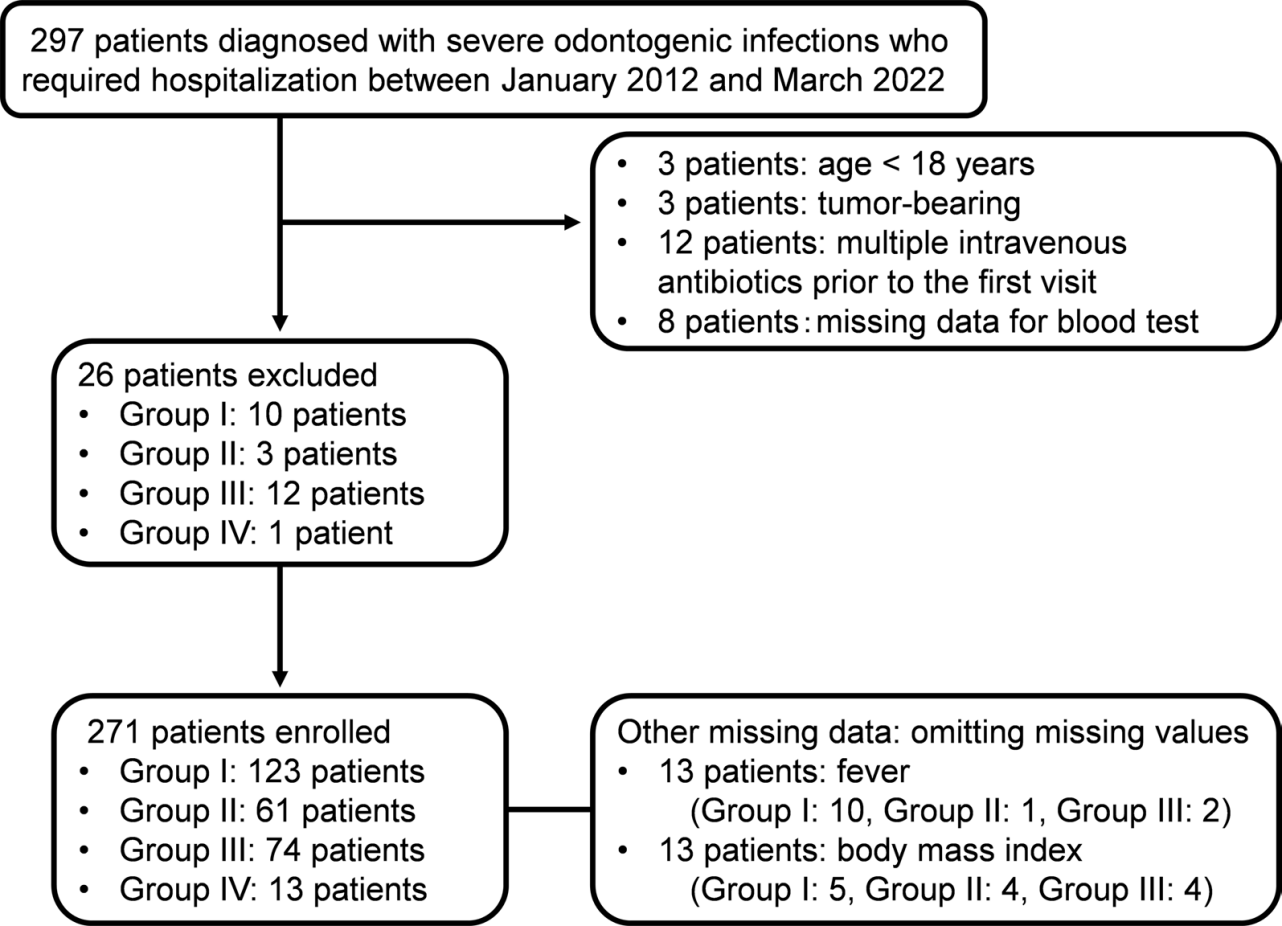
Flowchart of data collection and cleaning

There were 123 (45.4%), 61 (22.5%), 74 (27.3%), and 13 (4.8%) patients in Groups I, II, III, and IV, respectively (Fig. 1). CECT scans were performed in 108 patients (39.9%); 28 (22.8%) in Group I, 19 (31.2%) in Group II, 55 (74.3%) in Group III, and five (38.5%) in Group IV. Age, immunocompromised status, ICU admission, and duration of antimicrobial treatment tended to increase with increasing disease severity from Group I to Group IV (Table 1). The extent of opening was not necessarily less than the severity of the disease. Overall, 8.1% of patients were admitted to the ICU, and these patients exclusively belonged to groups III and IV. All the hematologic and inflammatory parameters showed an increasing trend from Group I to Group IV (*P* < 0.001); in contrast, Hb and lymphocyte fraction tended to decrease (Table 2). Among the various hematologic and inflammatory parameters, the LRINEC score was the parameter that best reflected ICU admission (cut-off 6, area under the curve [AUC] 0.92, sensitivity 77.3%, specificity 92.0%); CRP (cut-off 17.4, AUC 0.87, sensitivity 77.3%, specificity 88.8%), NLR (cut-off 10.1, AUC 0.85, sensitivity 86.4%, specificity 75.6%). For the bacterial culture results, the detection rate per group was 59.0% for Group II, 68.9% for Group III, and 61.5% for Group IV. *Streptococcus* species were the most common causative bacteria in all the groups, followed by *Parvimonas micra* and *Prevotella* species (Table 3). Obligate anaerobic bacteria were highly prevalent in Groups III and IV, and the median time required for culture identification was 7 days (range 2–19 days).

**Table 2:** Comparison of hematologic and inflammatory markers among groups

	Group				*P* value^a^	Trend test^b^
	I (n = 123)	II (n = 64)	III (n = 71)	IV (n = 13)		*P* value
Blood test data						
Albumin (g/dl)	3.8 [3.3, 4.2]	4.1 [3.6, 4.1]	3.4 [3.1, 3.7]	2.7 [2.4, 3.0]	< 0.001*	< 0.001*
C-reactive protein (mg/dl)	7.5 [4.4, 10.3]	9.2 [6.1, 13.1]	15.3 [10.1, 19.0]	23.9 [21.0, 32.3]	< 0.001*	< 0.001*
White blood cell (× 10^3^/µl)	10.8 [9.1, 13.3]	12.2 [9.9, 15.7]	14.3 [11.8, 16.6]	19.0 [14.7, 21.0]	< 0.001*	< 0.001*
Neutrophil (%)	77.1 [72.0, 82.1]	80.5 [74.5, 83.1]	86.5 [83.1, 88.2]	91.5 [89.4, 93.4]	< 0.001*	< 0.001*
Lymphocyte (%)	14.7 [11.1, 19.2]	12.7 [9.6, 16.8]	7.1 [5.0, 10.2]	3.9 [2.4, 6.0]	< 0.001*	< 0.001*
Platelet (× 10^4^/µl)	24.6 [20.7, 29.7]	25.7 [19.7, 31.4]	29.1 [22.9, 37.5]	33.1 [18.0, 36.6]	0.001*	< 0.001*
Hemoglobin (g/dl)	13.8 [12.3, 14.7]	13.4 [12.7, 15.1]	12.9 [11.6, 14.2]	11.9 [10.7, 13.1]	0.004*	0.004*
Sodium (mmol/L)	139 [137, 140]	138 [137, 140]	139 [137, 140]	136 [133, 140]	0.059	0.073
Creatinine (mg/dl)	0.75 [0.62, 0.92]	0.76 [0.62, 0.92]	0.74 [0.62, 1.01]	0.98 [0.77, 1.59]	0.091	0.188
Glucose (mg/dl)	110 [99, 120]	121 [100, 148]	116 [105, 132]	112 [96, 138]	0.083	0.073
LRINEC score	1 [0, 1]	1 [1, 2]	4 [1, 5]	7 [6, 8]	< 0.001*	< 0.001*
NLR	5.2 [3.8, 7.4]	6.3 [4.7, 8.6]	12.1 [8.0, 17.5]	23.3 [15.3, 38.9]	< 0.001*	< 0.001*
PLR	156.2 [126.8, 193.2]	166.5 [128.7, 232.3]	319.0 [224.4, 394.1]	301.9 [235.5, 662.4]	< 0.001*	< 0.001*
SII	134.4 [93.0, 177.4]	164.1 [107.9, 228.8]	349.1 [239.6, 533.3]	706.3 [494.7, 925.7]	< 0.001*	< 0.001*
CRP + NLR	13.4 [9.5, 16.6]	16.3 [12.5, 22.1]	27.8 [21.0, 35.2]	51.0 [35.1, 60.8]	< 0.001*	< 0.001*

Data are shown as the median [first quartile, third quartile]

^*^Statistically significant (*P* < 0.05)

^a^Kruskal − Wallis test; ^b^Jonckheere − Terpstra test

*LRINEC* laboratory risk indicator for necrotizing fasciitis, *NLR* neutrophil-to-lymphocyte ratio, *PLR* platelet-to-lymphocyte ratio, *SII* systemic immune-inflammation index

**Table 3:** Detection rate of pathogenic bacteria from an abscess or necrotic tissue by group

Group II (36/61)	No (%)	Group III (51/74)	No (%)	Group IV (8/13)	No (%)
[Facultative anaerobic]	33	[Facultative anaerobic]	36	[Facultative anaerobic]	10
*Streptococcus* spp.^a^	28 (84.8)	*Streptococcus* spp.^b^	31 (86.1)	*Streptococcus* spp.^c^	9 (90.0)
*Staphylococcus* spp.^d^	3 (9.1)	*Staphylococcus* spp.^e^	2 (5.6)	*Actinomyces* spp.	1 (10.0)
*Klebsiella pneumoniae*	1 (3.0)	*Actinomyces odontolyticus*	1 (2.8)		
*Haemophilus* spp.	1 (3.0)	*Lactobacillus casei*	1 (2.8)		
		*Propionibacterium acnes*	1 (2.8)		
[Obligate anaerobic]	17	[Obligate anaerobic]	43	[Obligate anaerobic]	11
*Parvimonas micra*	6 (35.3)	*Parvimonas micra*	18 (41.9)	*Prevotella* spp.^f^	6 (54.5)
*Prevotella* spp.^g^	4 (23.5)	*Prevotella* spp.^h^	10 (23.3)	*Parvimonas micra*	2 (18.2)
*Veillonella* spp.	3 (17.6)	*Peptostreptococcus* spp. ^i^	6 (14.0)	*Peptostreptococcus* spp. ^j^	2 (18.2)
*Peptostreptococcus* spp.	1 (5.9)	*Porphyromonas* spp.	3 (7.0)	*Eggerthella catenafoemis*	1 (9.1)
*Bacteroides* spp.	1 (5.9)	*Fusobacterium* spp. ^k^	3 (7.0)		
*Porphyromonas* spp.	1 (5.9)	*Anaerococcus* spp. ^l^	2 (4.7)		
*Fusobacterium nucleatum*	1 (5.9)	*Finegoldia magna*	1 (2.3)		

^a^*S. anginosus* (6); *S. constellatus* (5); *S. sanguinis* (2); *S. mitis* (2); *S. parasanguinis* (1); *S. gordonii* (1); *S. Salivarius* (1); unidentified species (10)

^b^*S. constellatus* (11); *S. anginosus* (8); *S. intermedius* (4); *S. mitis* (1); *S. sanguinis* (1); *S. gordonii* (1); unidentified species (5)

^c^*S. constellatus* (2); *S. anginosus* (2); *S. cristatus* (2); *S. salivarius* (2); *S. intermedius* (1)

^d^*S. aureus* (1); methicillin-resistant *S. aureus* (1); methicillin-susceptible S *aureus* (1)

^e^*S. aureus* (1); *S. hominis* (1)

^f^*P. Intermedia* (3); *P. buccae* (1); *P. Melaninogenica* (1); unidentified species (5)

^g^*P. intermedia* (3); *P. oris* (1)

^h^*P. intermedia* (3); *P. buccae* (2); *P. oris* (1); unidentified species (4)

^i^*P. anaerobius* (3); unidentified species (3)

^j^*P. asaccharolytics* (1); *P. anaerobius* (1)

^k^*F. nucleatum* (2); unidentified species (1)

^l^*A. prevotii* (1); unidentified species (1)

### *Groups I* + *II* + *III versus group IV*

**Fig. 2:**
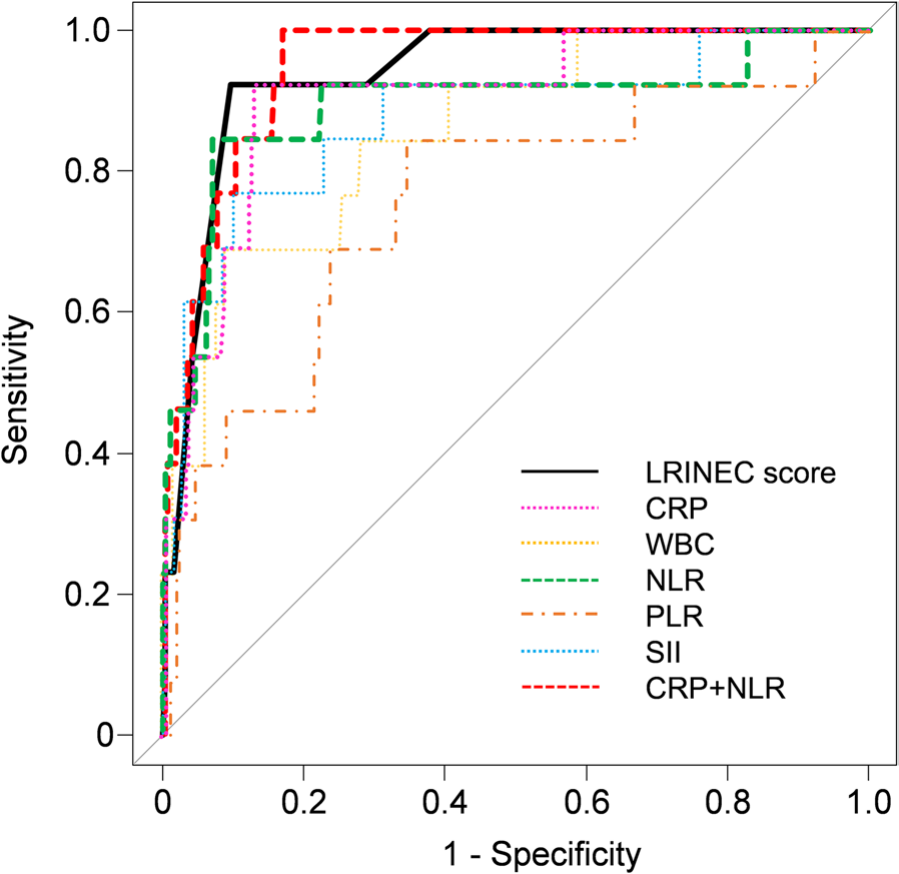
ROC analysis for the diagnostic performance of each of the hematologic and inflammatory parameters for NSTI. *ROC* receiver operating characteristic, *NTSI* necrotizing soft tissue infection, *LRINEC* laboratory risk indicator for necrotizing fasciitis, *CRP* C-reactive protein, *WBC* White blood cell, *NLR* Neutrophil-to-lymphocyte ratio, *PLR* Platelet-to-lymphocyte ratio, *SII* systemic immune-inflammation index

This comparison was made to avoid oversight of NSTI diagnosis. Group IV showed significantly lower alb and Hb values than the other groups (*P* < 0.001, *P* = 0.027). All hematologic and inflammatory parameters were significantly higher in Group IV (Table 4). The ROC analysis for hematologic and inflammatory parameters as predictive tests for NSTI showed that the LRINEC score, NLR, and CRP + NLR had an AUC of > 0.9. Moreover, the negative predictive value (NPV) was generally high for all hematologic and inflammatory parameters (Fig. 2, Table 5).

**Table 4:** Comparison of characteristics and hematologic and inflammatory parameters between Groups I + II + III and IV

Group	I + II + III (n = 258)	IV (n = 13)	*P* value
Age (years)	61 [41, 74]	73 [41, 75]	0.298
Sex (male)	133 (51.6%)	5 (38.5%)	0.405
Body mass index	22.4 [20.2, 25.0]	21.3 [18.7, 23.4]	0.345
Cause			0.672
Lesion			0.571
Maxilla	53 (20.5%)	1 (7.7%)	
Mandible	200 (77.5%)	12 (92.3%)	
Others^†^	5 (1.9%)	0	
Location of odontogenic cause			0.565
Anterior	32 (12.4%)	1 (7.7%)	
Premolar	20 (7.8%)	0	
Molar	180 (69.8%)	12 (92.3%)	
Others^‡^	26 (10.1%)	0	
Compromised host^§^	63 (24.4%)	7 (53.8%)	0.044*
Fever (°C)	37.2 [36.6, 37.7]	37.4 [36.9, 37.8]	0.207
Blood test data			
Albumin (g/dl)	3.6 [3.3, 4.0]	2.7 [2.4, 3.0]	< 0.001*
C-reactive protein (mg/dl)	9.1 [5.7, 13.9]	23.9 [21.0, 32.3]	< 0.001*
White blood cell (× 10^3^/µl)	12.1 [9.7, 14.7]	19.0 [14.7, 21.0]	< 0.001*
Neutrophil (%)	80.8 [74.7, 85.4]	91.5 [89.4, 93.4]	< 0.001*
Lymphocyte (%)	12.0 [8.1, 17.0]	3.9 [2.4, 6.0]	< 0.001*
Platelet (× 10^4^/µl)	26.2 [21.1, 31.5]	33.1 [18.0, 36.6]	0.382
Hemoglobin (g/dl)	13.4 [12.3, 14.6]	11.9 [10.7, 13.1]	0.027*
Sodium (mmol/L)	139 [137, 140]	136 [133, 140]	0.064
Creatinine (mg/dl)	0.76 [0.62, 0.94]	0.98 [0.77, 1.59]	0.035*
Glucose (mg/dl)	115 [100, 133]	112 [96, 138]	0.988
LRINEC score	1 [1, 4]	7 [6, 8]	< 0.001*
NLR	6.7 [4.4, 10.3]	23.3 [15.3, 38.9]	< 0.001*
PLR	179.8 [134.3, 265.3]	301.9 [235.5, 662.4]	0.006*
SII	173.7 [110.2, 292.3]	706.3 [494.7, 925.7]	< 0.001*
CRP + NLR	16.3 [12.0, 23.8]	51.0 [35.1, 60.8]	< 0.001*

Data are shown as the median [first quartile, third quartile]

^*^Statistically significant (*P* < 0.05)

*LRINEC* laboratory risk indicator for necrotizing fasciitis, *NLR*, neutrophil-to-lymphocyte ratio; PLR, platelet-to-lymphocyte ratio; SII, systemic immune-inflammation index

^†^Buccal; tongue

^‡^Maxillary sinus; salivary gland; lip

^§^Diabetes mellitus; corticosteroids usage; hemodialysis

**Table 5:** Comparison of hematologic and inflammatory parameters as predictive tests for necrotizing soft tissue infection

	Cut-off value	AUC	95% CI	Sensitivity (%)	Specificity	PPV	NPV (%)	LR +	LR-
CRP (mg/dl)	20.9	0.894	0.771–1.000	84.6	93.0%	37.9%	99.2	12.1	0.17
WBC (× 10^3^/µl)	17.5	0.857	0.754–0.961	69.2	91.1%	28.1%	98.3	7.76	0.33
LRINEC score	6	0.941	0.890–0.992	92.3	90.3%	32.4%	99.6	9.53	0.09
NLR	14.2	0.901	0.817–0.985	92.3	86.8%	26.1%	99.6	7.00	0.09
PLR	229	0.758	0.605–0.911	84.6	65.5%	11.0%	98.8	2.45	0.23
SII	495	0.870	0.754–0.986	76.9	89.5%	27.0%	98.7	7.35	0.26
CRP + NLR	27	0.947	0.910–0.948	100	82.6%	22.4%	100	5.73	0

*CRP* C-reactive protein, *WBC* white blood cell, *LRINEC* laboratory risk indicator for necrotizing fasciitis, *NLR* neutrophil-to-lymphocyte ratio, *PLR* platelet-to-lymphocyte ratio, *SII* systemic immune-inflammation index, *AUC* area under the curve, *CI* confidence interval, *PPV* positive predictive value, *NPV* negative predictive value, *LR + * positive likelihood ratio, *LR−* negative likelihood ratio

### *Groups I* + *II versus groups III* + *IV*

Groups III and IV require early detection and surgical treatment (drainage and debridement), and a CECT image is considered effective. This comparison was made for decision-making to perform CECT imaging. All hematologic and inflammatory parameters were significantly higher in Groups III + IV (Additional file 1: Table S1). In this study, all patients underwent urgent incisional drainage and debridement immediately after diagnosis in Groups III and IV.

### Decision tree analysis

**Fig. 3:**
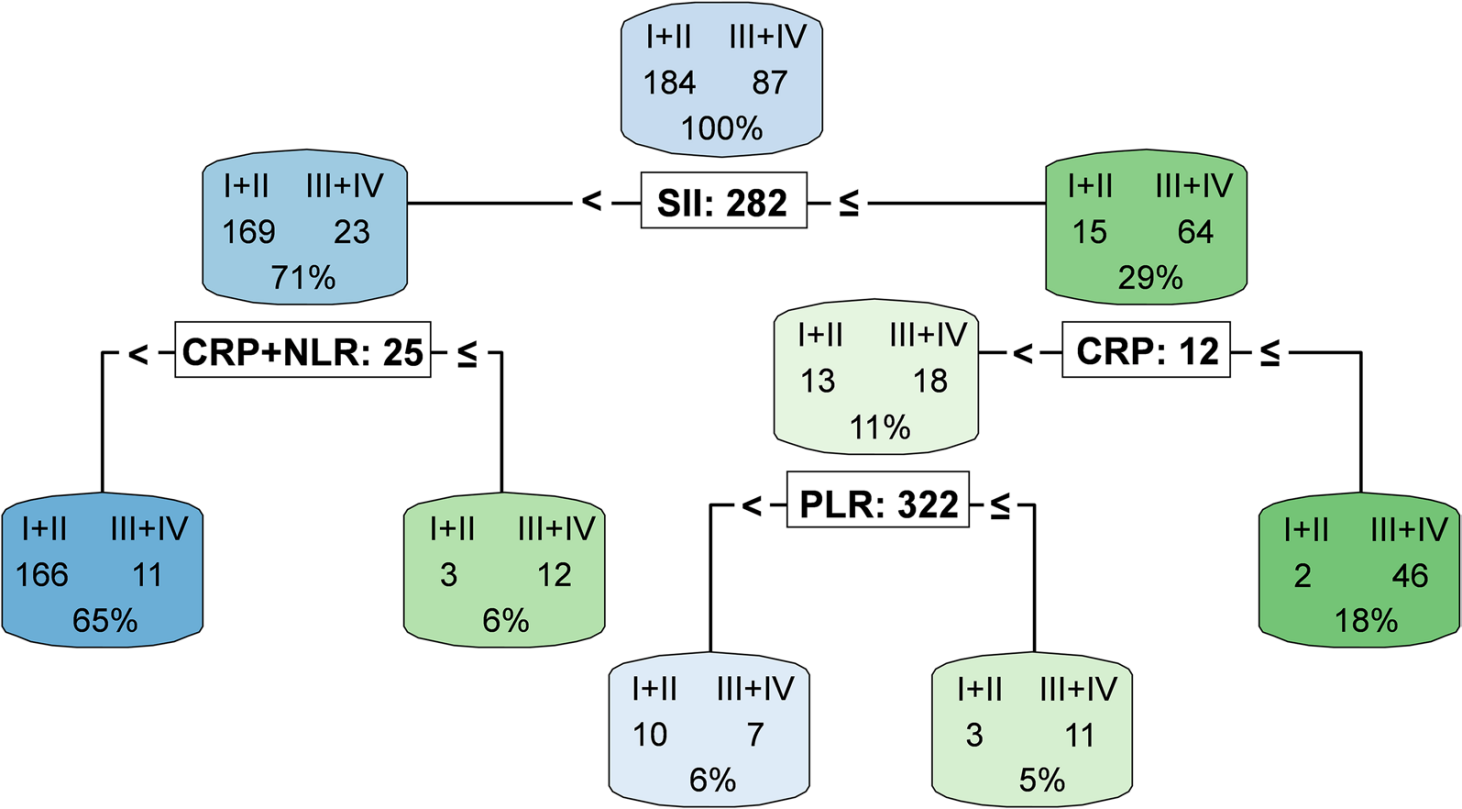
Decision tree analysis for discrimination between Groups I + II and Groups III + IV. The node (the frame) shows the group (I + II, III + IV), the number of cases, as well as the proportion of the total data. If the element (split condition expression) is satisfied, it goes left (node); if not, to the right. The color of the node indicates “I + II > III + IV” for blue and “III + IV > I + II” for green. The darker the node’s color, the lower its entropy (average information content); that is, the higher its purity. For example, if a node is dark green, it indicates that the node is mostly III + IV, indicating high classification accuracy. Note that the one order of magnitude for SII is smaller than that of the original. Group I: cellulitis; Group II: cellulitis with shallow abscess formation; Group III: deep neck abscess; and Group IV: necrotizing soft tissue infection. *CRP* C-reactive protein, *NLR* neutrophil-to-lymphocyte ratio, *PLR* platelet-to-lymphocyte ratio; SII, systemic immune-inflammation index

According to the trend test, almost all hematologic and inflammatory parameters were found to increase as the group progressed (i.e., Group I < Group II < Group III < Group IV). Also, all parameters were significantly higher in Groups III + IV than in Groups I + II. Subsequently, to distinguish as accurately as possible between Group III + IV and Group I + II, we attempted to calculate specific values for each parameter combination using decision tree analysis. Seven explanatory variables were used in the analysis: the LRINEC score, existing hematologic and inflammatory parameters (WBC, CRP, NLR, and PLR), and new markers (SII and CRP + NLR) (Fig. 3). The most important variable was SII, followed by CRP + NLR, PLR, NLR, CRP, WBC, and the LRINEC scores. There were 79 patients with SII of ≥ 282: 15 patients (8.2%) in Groups I + II and 64 patients (73.6%) in Groups III + IV. In total, 166 patients (90.2%) in Groups I + II and 11 patients (12.6%) in Groups III + IV had SII < 282 and CRP + NLR < 25. The classification accuracy was 89.3%, and abscess formation was clinically suspected due to touching waves for the latter 11 patients (all Group III).

Overall, 18 patients did not fit the analysis in Groups I + II. Nine patients in Group II had abscess formation over a wide area. In the remaining nine patients, the inflammation (not abscess) spilled over into the deep anatomic space (submandibular, pterygomandibular, and parapharyngeal space) for five patients, three of whom had sepsis, and one patient had a halfway incision for abscess formation by the previous physician.

## Discussion

This study found that almost all hematologic and inflammatory parameters increased with progressing severity of odontogenic infection; both CRP + NLR (with a cut-off of 27) and LRINEC score (with a cut-off of 6) are effective adjunct diagnostic tools for NSTI. This means that the severity of odontogenic infections can almost be evaluated by blood tests. In addition, CRP + NLR and SII are effective adjunct parameters in decision-making for CECT imaging. To the best of our knowledge, this is the first report using hematologic and inflammatory parameters, especially SII and CRP + NLR, to assess the severity of bacterial infections in the head and neck region, including NSTI. In this study, *Streptococcus* species and anaerobes were found to be the most common causative organisms of severe odontogenic infections, with anaerobes becoming increasingly involved, especially in more severe cases.

CRP is often used as a marker of inflammation and is reported to be useful for detecting infections in the head and neck region [21–23]. However, it does not strictly reflect real-time disease status as it peaks approximately 2 days after infection onset [19]. A previous report investigated the efficacy of NLR compared to CRP for deep neck infections associated with odontogenic infections [24]. The cut-off values of NLR and CRP in patients who required ICU admission were 11.75 (sensitivity 66.7%, specificity 82.6%, AUC 0.766) and 18 mg/dl (sensitivity 66.7%, specificity 85.5%, AUC 0.815), respectively. In this study, the same was largely true. In addition, NLR and CRP were also higher in more severe cases, and the cut-off value for NSTI was considered reasonable (NLR 14.2, CRP 20.9 mg/dl). Contrarily, there are no reports evaluating the efficacy of CRP + NLR for infectious diseases. The results of this study suggest that CRP + NLR could more accurately reflect the severity of bacterial odontogenic infections than CRP or NLR.

NSTI remains a fatal disease, with an approximately 20% mortality rate [25]; thus, it requires immediate debridement and antimicrobial administration [26, 27]. However, 35–85% of cases of NSTI are misdiagnosed as cellulitis or abscess at the initial presentation [28, 29]. This study confirmed the validity of the LRINEC score; however, a previous study reported that it was not useful for identifying infections in the head-neck region [30]. Recent meta-analyses also reported that the LRINEC score could not reliably rule out NSTI [6, 31]. The components of the LRINEC score were suggested to be inevitably higher in diabetic renal failure and other conditions. Similarly, a study reported a case of necrotizing fasciitis with an LRINEC score of 0 [32]. Meanwhile, the CRP + NLR score devised in this study may be easier to use than the LRINEC score because it is less susceptible to the influence of certain pathological conditions, except for hematological diseases. Based on the results of this study, the calculation of CRP + NLR together with the LRINEC score could help in the diagnosis of NSTI.

Deep neck abscesses have been associated with complications such as sepsis, airway obstruction, mediastinitis, internal jugular venous thrombosis, and carotid artery rupture [33–35]. Therefore, early detection and treatment are necessary. CECT images are useful for detecting abscesses, including NSTI [6, 7, 36]. However, radiation exposure, the risk of allergy (anaphylactic shock), and other factors (e.g., biguanide use for diabetes mellitus, impaired renal function, asthma, and thyroid dysfunction) must be considered. Therefore, CECT imaging should not be routinely performed for severe odontogenic infections. When struggling to decide to perform CECT, the results of this study suggest that SII and CRP + NLR could be useful in decision-making.

There were also high values for SII or CRP + NLR in Groups I and II, which were associated with sepsis, shallow but extensive abscess formation, and inflammation that spilled into the deep anatomic space. Therefore, the combination of CRP + NLR and SII was considered to almost accurately reflect the severity of the infection and the relative degree of inflammation. Concurrently, in this study, 11 patients had deep neck abscesses who did not fit the decision tree analysis. In these patients, clinical findings led to the suspicion of abscess formation; therefore, CECT imaging was performed. Thus, clinical findings are crucial, and the algorithm based on decision tree analysis should only be used as an adjunct.

The causative organisms identified in this study were similar to those in previous reviews [37–39]. Although there was no difference in the species of bacteria in each condition, anaerobic bacteria were detected in a higher percentage of NSTI and deep neck abscess cases. As stated in previous reports, we reaffirmed the need to target streptococci and obligate anaerobes in empiric therapy.

In this study, the criteria for requiring hospitalization were established based on clinical findings, and there are no clear guidelines. There is a report using a scoring system for decision-making to hospitalize [40], which is similar to the concept of this study. In the future, we would consider creating new diagnostic tools including criteria for admission.

### Limitations

First, observer and recorder bias may have been introduced during data collection owing to the retrospective design; to reduce this bias, data were collected and recorded by three independent observers. Second, patients with conditions that could modify blood test results and infectious states (e.g., those on anticancer treatments and multiple intravenous antibiotics) were excluded from the analysis; more accurate indicators would be needed to accommodate such patients. Third, this study was based at a single facility, and differences may occur among facilities depending on regional characteristics and measurement instruments; consequently, collaborative research at other facilities is desirable.

## Conclusions

Hematologic and inflammatory parameters in blood tests are useful as adjunctive diagnostic parameters for NSTI and deep neck abscesses, although clinical symptoms remain paramount. Especially, the cut-off value of 27 for CRP + NLR, along with the LRINEC score of 6, was useful to exclude the diagnosis of NSTI. In addition, SII and CRP + NLR help diagnose NSTI and deep neck abscesses, thus supporting decision-making for CECT imaging, with (1) SII ≥ 282 or (2) SII < 282 and CRP + NLR ≥ 25 indicating an aggressive need for CECT. In the future, multicenter prospective studies are required to confirm our findings. In addition, a more accurate indicator that can be used in the early detection of fatal bacterial infections in the head and neck region for patients from diverse backgrounds is desired.

## Supplementary Information

**Additional file 1: Figure S1.** The relationship between age and odontogenic causes. **Table S1.** Comparison of characteristics, blood test data, and hematologic and inflammatory parameters between Groups I+II and III+IV.

## Data Availability

The datasets used and analyzed during the current study are available from the corresponding author upon reasonable request.

## Abbreviations

NSTI: Necrotizing soft tissue infection
CECT: Contrast-enhanced computed tomography
CRP: C-reactive protein
WBC: White blood cell
LRINEC: Laboratory risk indicator for necrotizing fasciitis
NLR: Neutrophil-to-lymphocyte ratio
PLR: Platelet-to-lymphocyte ratio
SII: Systemic immune-inflammation index
ICU: Intensive care unit
Plt: Platelet
Na: Sodium
Hb: Hemoglobin
Glu: Glucose
ROC: Receiver operating characteristic
AUC: Area under the curve
NPV: Negative predictive value

## References

[1] Huang TT, Liu YC, Chen PR, Tseng FY, Yeh TH, Chen YS (2004). Deep neck infection: analysis of 185 cases. Head Neck.

[2] Zemplenyi K, Lopez B, Sardesai M, Dillon JK (2017). Can progression of odontogenic infections to cervical necrotizing soft tissue infections be predicted?. Int J Oral Maxillofac Surg.

[3] Prabhu SR, Nirmalkumar ES (2019). Acute fascial space infections of the neck: 1034 cases in 17 years follow up. Ann Maxillofac Surg.

[4] Grisaru-Soen G, Komisar O, Aizenstein O, Soudack M, Schwartz D, Paret G (2010). Retropharyngeal and parapharyngeal abscess in children—epidemiology, clinical features and treatment. Int J Pediatr Otorhinolaryngol.

[5] Saluja S, Brietzke SE, Egan KK, Klavon S, Robson CD, Waltzman ML (2013). A prospective study of 113 deep neck infections managed using a clinical practice guideline. Laryngoscope.

[6] Fernando SM, Tran A, Cheng W, Rochwerg B, Kyeremanteng K, Seely AJE (2019). Necrotizing soft tissue infection: diagnostic accuracy of physical examination, imaging, and LRINEC score: a systematic review and meta-analysis. Ann Surg.

[7] Cunqueiro A, Gomes WA, Lee P, Dym RJ, Scheinfeld MH (2019). CT of the neck: image analysis and reporting in the emergency setting. Radiographics.

[8] Wong CH, Khin LW, Heng KS, Tan KC, Low CO (2004). The LRINEC (laboratory risk indicator for necrotizing fasciitis) score: a tool for distinguishing necrotizing fasciitis from other soft tissue infections. Crit Care Med.

[9] Iwata E, Kusumoto J, Takata N, Furudoi S, Tachibana A, Akashi M (2021). The characteristics of oro-cervical necrotizing fasciitis-comparison with severe cellulitis of oro-cervical region and necrotizing fasciitis of other body regions. PLoS ONE.

[10] Zahorec R (2001). Ratio of neutrophil to lymphocyte counts—rapid and simple parameter of systemic inflammation and stress in critically ill. Bratisl Lek Listy.

[11] Smith RA, Ghaneh P, Sutton R, Raraty M, Campbell F, Neoptolemos JP (2008). Prognosis of resected ampullary adenocarcinoma by preoperative serum CA19-9 levels and platelet–lymphocyte ratio. J Gastrointest Surg.

[12] Baglam T, Binnetoglu A, Yumusakhuylu AC, Gerin F, Demir B, Sari M (2015). Predictive value of the neutrophil-to-lymphocyte ratio in patients with deep neck space infection secondary to acute bacterial tonsillitis. Int J Pediatr Otorhinolaryngol.

[13] Liu L, Shao Z, Yu H, Zhang W, Wang H, Mei Z (2020). Is the platelet to lymphocyte ratio a promising biomarker to distinguish acute appendicitis? Evidence from a systematic review with meta-analysis. PLoS ONE.

[14] Hu B, Yang XR, Xu Y, Sun YF, Sun C, Guo W (2014). Systemic immune-inflammation index predicts prognosis of patients after curative resection for hepatocellular carcinoma. Clin Cancer Res.

[15] Levitt GW (1970). Cervical fascia and deep neck infections. Laryngoscope.

[16] Fisher JR, Conway MJ, Takeshita RT, Sandoval MR (1979). Necrotizing fasciitis Importance of roentgenographic studies for soft-tissue gas. JAMA.

[17] Mathieu D, Neviere R, Teillon C, Chagnon JL, Lebleu N, Wattel F (1995). Cervical necrotizing fasciitis: clinical manifestations and management. Clin Infect Dis.

[18] Honda T, Uehara T, Matsumoto G, Arai S, Sugano M (2016). Neutrophil left shift and white blood cell count as markers of bacterial infection. Clin Chim Acta.

[19] Pepys MB, Hirschfield GM (2003). C-reactive protein: a critical update. J Clin Invest.

[20] The Japanese Association for Infectious Disease (2018). The 2016 JAID/JSC guidelines for clinical management of infectious disease–Odontogenic infections. J Infect Chemother..

[21] Stathopoulos P, Igoumenakis D, Shuttleworth J, Smith W, Ameerally P (2017). Predictive factors of hospital stay in patients with odontogenic maxillofacial infections: the role of C-reactive protein. Br J Oral Maxillofac Surg.

[22] Akashi M, Furudoi S, Hashikawa K, Sakakibara A, Hasegawa T, Shigeta T (2015). Postoperative abnormal response of C-reactive protein as an indicator for infectious complications after oral oncologic surgery with primary reconstruction. J Otolaryngol Head Neck Surg.

[23] Heim N, Wiedemeyer V, Reich RH, Martini M (2018). The role of C-reactive protein and white blood cell count in the prediction of length of stay in hospital and severity of odontogenic abscess. J Craniomaxillofac Surg.

[24] Gallagher N, Collyer J, Bowe CM (2021). Neutrophil to lymphocyte ratio as a prognostic marker of deep neck space infections secondary to odontogenic infection. Br J Oral Maxillofac Surg.

[25] Ord R, Coletti D (2009). Cervico-facial necrotizing fasciitis. Oral Dis.

[26] Stevens DL, Bisno AL, Chambers HF, Dellinger EP, Goldstein EJC, Gorbach SL (2014). Practice guidelines for the diagnosis and management of skin and soft tissue infections: 2014 update by the infectious diseases society of America. Clin Infect Dis.

[27] Liu YM, Chi CY, Ho MW, Chen CM, Liao WC, Ho CM (2005). Microbiology and factors affecting mortality in necrotizing fasciitis. J Microbiol Immunol Infect.

[28] Haywood CT, McGeer A, Low DE (1999). Clinical experience with 20 cases of group A streptococcus necrotizing fasciitis and myonecrosis: 1995 to 1997. Plast Reconstr Surg.

[29] Wong CH, Chang HC, Pasupathy S, Khin LW, Tan JL, Low CO (2003). Necrotizing fasciitis: clinical presentation, microbiology, and determinants of mortality. J Bone Joint Surg Am.

[30] Thomas AJ, Meyer TK (2012). Retrospective evaluation of laboratory-based diagnostic tools for cervical necrotizing fasciitis. Laryngoscope.

[31] Kim DH, Kim SW, Hwang SH (2022). Application of the laboratory risk indicator for necrotizing fasciitis score to the head and neck: a systematic review and meta-analysis. ANZ J Surg.

[32] Wilson MP, Schneir AB (2013). A case of necrotizing fasciitis with a LRINEC score of zero: clinical suspicion should trump scoring systems. J Emerg Med.

[33] Gidley PW, Ghorayeb BY, Stiernberg CM (1997). Contemporary management of deep neck space infections. Otolaryngol Head Neck Surg.

[34] Hasegawa J, Hidaka H, Tateda M, Kudo T, Sagai S, Miyazaki M (2011). An analysis of clinical risk factors of deep neck infection. Auris Nasus Larynx.

[35] Weise H, Naros A, Weise C, Reinert S, Hoefert S (2019). Severe odontogenic infections with septic progress – a constant and increasing challenge: a retrospective analysis. BMC Oral Health.

[36] Rosenthal M, Oreadi D, Kraus J, Bedi H, Stark PC, Shastri K (2011). Comparison of preoperative computed tomography and surgical findings in maxillofacial infections. J Oral Maxillofac Surg.

[37] Poeschl PW, Spusta L, Russmueller G, Seemann R, Hirschl A, Poeschl E (2010). Antibiotic susceptibility and resistance of the odontogenic microbiological spectrum and its clinical impact on severe deep space head and neck infections. Oral Surg Oral Med Oral Pathol Oral Radiol Endod.

[38] Gao W, Lin Y, Yue H, Chen W, Liu T, Ye J (2022). Bacteriological analysis based on disease severity and clinical characteristics in patients with deep neck space abscess. BMC Infect Dis.

[39] Velhonoja J, Lääveri M, Soukka T, Irjala H, Kinnunen I (2020). Deep neck space infections: an upward trend and changing characteristics. Eur Arch Otorhinolaryngol.

[40] Sainuddin S, Hague R, Howson K, Clark S (2017). New admission scoring criteria for patients with odontogenic infections: a pilot study. Br J Oral Maxillofac Surg.

