# Supplementary material for: Hematologic and inflammatory parameters for determining severity of odontogenic infections at admission: a retrospective study
Source: BMC Infect Dis. 2022 Dec 12;22:931. doi: 10.1186/s12879-022-07934-x (PMC9743669; doi:10.1186/s12879-022-07934-x)
Supplement: Supplementary file 1 — Additional file 1: Figure S1. The relationship between age and odontogenic causes. Table S1. Comparison of characteristics, blood test data, and hematologic and inflammatory parameters between Groups I+II and III+IV. [file 12879_2022_7934_MOESM1_ESM.docx]

**Additional file 1**

**Hematologic and Inflammatory Parameters for Determining Severity of Odontogenic Infections at Admission: A retrospective study**

Junya Kusumoto^1,2^*, Eiji Iwata^1^, Wensu Huang^1,3^, Naoki Takata^1,4^, Akira Tachibana^1^, Masaya Akashi^2^

^1^ Department of Oral and Maxillofacial Surgery, Kakogawa Central City Hospital, Kakogawa, Japan

^2^ Department of Oral and Maxillofacial Surgery, Kobe University Graduate School of Medicine, Kobe, Japan

^3^ Department of Oral and Maxillofacial Surgery, Mitsubishi Kobe Hospital, Kobe, Japan

^4^ Department of Oral and Maxillofacial Surgery, Hyogo Prefectural Awaji Medical Center, Awaji, Japan

*Correspondence and reprint requests should be addressed to:

Junya Kusumoto

Department of Oral and Maxillofacial Surgery, Kobe University Graduate School of Medicine

7-5-2, Kusunoki-cho Chuo-ku, Kobe 650-0017, Japan

Telephone: +81-78-382-6213

E-mail: chivalry_2727@people.kobe-u.ac.jp

**Supplementary Figure**

**Figure S1 The relationship between age and odontogenic causes**

Pericoronitis was more common in younger patients. Osteomyelitis, periodontitis, and trauma were common in the elderly. Apical periodontitis and post-extraction infection were common in all age groups. In this study, post-extraction infection was included in SSI.

Apical periodontitis (44.4%, 56 [35.8, 75.3]); Pericoronitis (13.3%, 46 [33.8, 61]); Osteomyelitis (11.5%, 80 [70.5, 87.5]); Post-extraction infection (8.5%, 53 [32.5, 73]); Periodontitis (7.0%, 70 [61, 71.5]); Odontogenic cyst (4.8%, 53 [45, 65]); Sialadenitis (4.8%, 67 [56, 72]); Trauma (2.6%, ); Odontogenic maxillary sinusitis (1.9%, 46 [42, 53]); Foreign body (0.7%, mean 65.5); Surgical site infection (0.7%, mean 64.5)

(proportion, median [first quartile, third quartile])

**Supplementary Table**

**Table S1. Comparison of characteristics, blood test data, and hematologic and inflammatory parameters between Groups I+II and III+IV**

|  | I+II (n = 184) | III+IV (n = 87) | *P* value |
| --- | --- | --- | --- |
| Age (years) | 57.5 [37, 74.3] | 67 [46, 74.5] | 0.015* |
| Sex (male) | 95 (51.6%) | 43 (49.4%) | 0.795 |
| Body mass index | 22.6 [20.8, 25.2] | 21.3 [18.7, 24.6] | 0.009* |
| Cause |  |  | 1.000 |
| Lesion |  |  | < 0.001* |
| Maxilla | 47 (25.5%) | 7 (8.0%) |  |
| Mandible | 133 (72.3%) | 79 (90.8%) |  |
| Others† | 4 (2.2%) | 1 (1.2%) |  |
| Location of odontogenic cause |  |  | 0.042* |
| Anterior | 26 (14.1%) | 7 (8.0%) |  |
| Premolar | 18 (9.8%) | 2 (2.3%) |  |
| Molar | 122 (66.3%) | 70 (80.5%) |  |
| Others‡ | 18 (9.8%) | 8 (9.2%) |  |
| Compromised host^§^ | 42 (22.8%) | 28 (32.2%) | 0.105 |
| Fever (°C) | 37.1 [36.6, 37.7] | 37.4 [36.7, 37.7] | 0.440 |
| Blood test data |  |  |  |
| Albumin (g/dl) | 3.9 [3.5, 4.2] | 3.3 [2.9, 3.7] | < 0.001* |
| C-reactive protein (mg/dl) | 8.0 [4.8, 10.8] | 16.3 [10.8, 22.1] | < 0.001* |
| White blood cell (× 10^3^/µl) | 11.0 [9.3, 13.8] | 14.6 [12.2, 17.5] | < 0.001* |
| Neutrophil (%) | 78.4 [72.2, 82.1] | 86.8 [83.6, 90.1] | < 0.001* |
| Lymphocyte (%) | 13.9 [10.5, 18.4] | 6.7 [4.8, 9.8] | < 0.001* |
| Platelet (× 10^4^/µl) | 25.3 [20.1, 30.1] | 29.4 [22.6, 37.4] | < 0.001* |
| Hemoglobin (g/dl) | 13.7 [12.5, 14.8] | 12.8 [11.6, 14.2] | < 0.001* |
| Sodium (mmol/L) | 139 [137, 140] | 138 [136, 140] | 0.320 |
| Creatinine (mg/dl) | 0.76 [0.62, 0.92] | 0.77 [0.63, 1.02] | 0.229 |
| Glucose (mg/dl) | 114 [99, 133] | 116 [104, 134] | 0.329 |
| LRINEC score | 1 [0, 2] | 4 [1, 6] | < 0.001* |
| NLR | 5.7 [3.9, 8.0] | 13.1 [8.6, 18.3] | < 0.001* |
| PLR | 161 [127, 199] | 319 [227, 406] | < 0.001* |
| SII | 139 [95, 190] | 406 [257, 573] | < 0.001* |
| CRP+NLR | 14.5 [10.1, 18.1] | 28.8 [22.9, 40.8] | < 0.001* |

Data are shown as the median [first quartile, third quartile].

*Statistically significant (*P* < 0.05)

LRINEC, laboratory risk indicator for necrotizing fasciitis; NLR, neutrophil-to-lymphocyte ratio; PLR, platelet-to-lymphocyte ratio; SII, systemic immune-inflammation index

†, buccal; tongue

‡, maxillary sinus; salivary gland; lip

§, diabetes mellitus; corticosteroids usage; hemodialysis
